# Supplementary material for: Transforming brownfields into urban greenspaces: A working process for stakeholder analysis
Source: PLoS One. 2023 Jan 5;18(1):e0278747. doi: 10.1371/journal.pone.0278747 (PMC9815653; doi:10.1371/journal.pone.0278747)
Supplement: S1 File — (PDF) [file pone.0278747.s001.pdf]

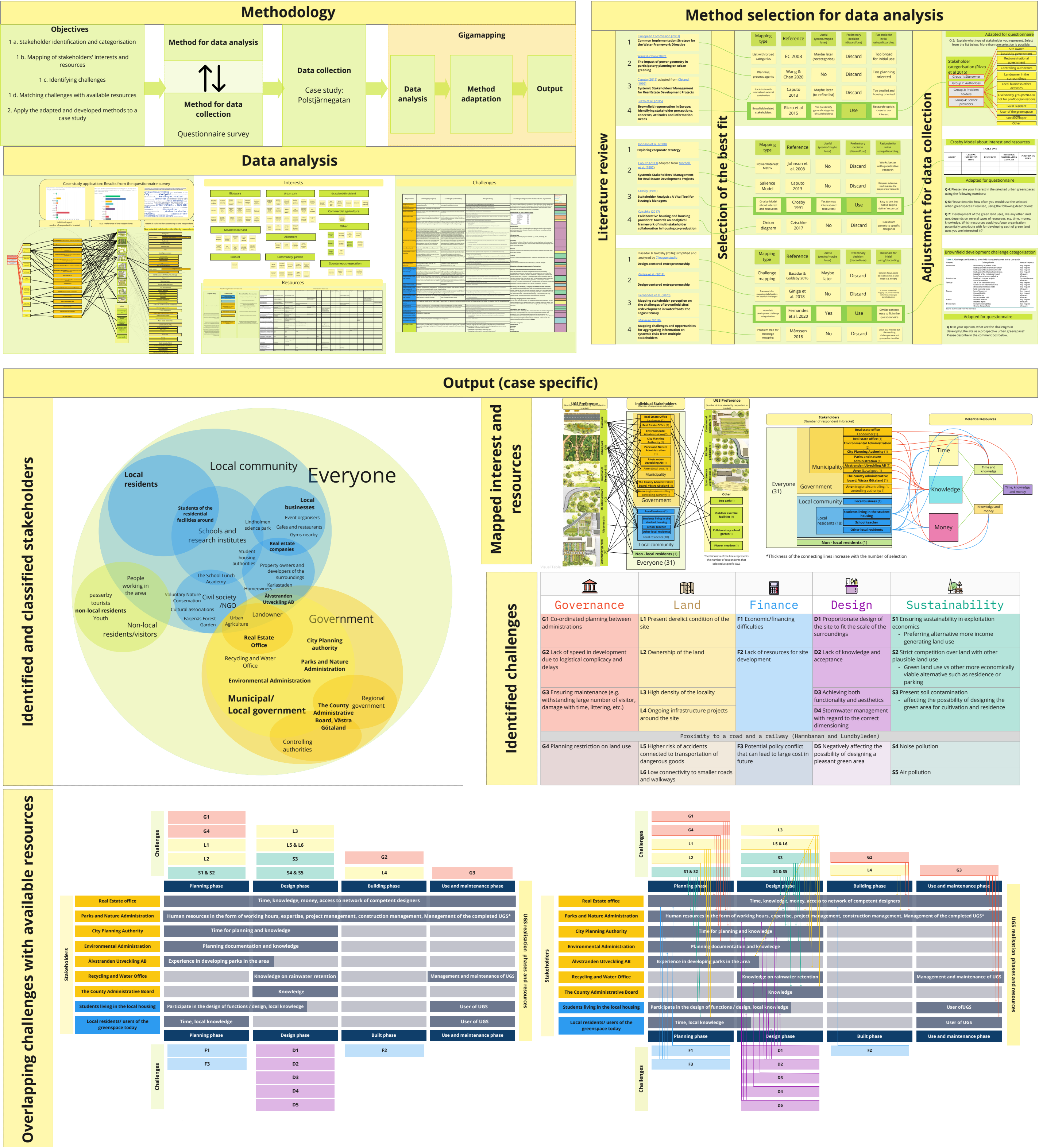

Methodology
Objectives
1 a. Stakeholder identification and categorisation
1 b. Mapping of stakeholders' interests and resources
1 c. Identifying challenges
1 d. Matching challenges with available resources
2. Apply the adapted and developed methods to a case study
Method for data analysis
Method for data collection
Questionnaire survey
Data collection
Case study: Polstjärnegatan
Gigamapping
Data analysis
Method adaptation
Output
Data analysis
Case study application: Results from the questionnaire survey
Interests
Resources
Challenges
Method selection for data analysis
Literature review
Selection of the best fit
Adjustment for data collection
Mapping type
Reference
Useful (yes/no/maybe later)
Preliminary decision (discard/use)
Rationale for initial using/discarding
European Commission (2003) Common Implementation Strategy for the Water Framework Directive
Wang & Chen (2020) The impact of power-geometry in participatory planning on urban greening
Caputo (2013) adapted from Cleland (1999) Systemic Stakeholders' Management for Real Estate Development Projects
Rizzo et al. (2015) Brownfield regeneration in Europe: Identifying stakeholder perceptions, concerns, attitudes and information needs
List with broad categories
EC 2003
Maybe later (recategorise)
Discard
Too broad for initial use
Planning process agents
Wang & Chan 2020
No
Discard
Too planning oriented
Caputo 2013
Maybe later (to refine list)
Discard
Too detailed and housing oriented
Brownfield related stakeholders
Rizzo et al 2015
Use
Research topic is close to our interest
Johnson et al. (2008) Exploring corporate strategy
Caputo (2013) adapted from Mitchell et al. (1997) Systemic Stakeholders' Management for Real Estate Development Projects
Crosby (1991) Stakeholder Analysis: A Vital Tool for Strategic Managers
Czischke (2017) Collaborative housing and housing providers: towards an analytical framework of multi-stakeholder collaboration in housing co-production
Power/Interest Matrix
Johnson et al. 2008
No
Discard
Works better with quantitative research
Salience Model
Caputo 2013
No
Discard
Crosby Model about interest and resources
Crosby 1991
Yes (to map interest and resources)
Use
Easy to use, but not so easy to define "resources"
Onion diagram
Czischke 2017
No
Discard
Goes from generic to specific categories
Basadur & Goldsby (2016); simplified and analysed by 7 league studio Design-centered entrepreneurship
Ginige et al. (2018) Design-centered entrepreneurship
Fernandes et al. (2020) Mapping stakeholder perception on the challenges of brownfield sites' redevelopment in waterfronts: the Tagus Estuary
Månsson (2018) Mapping challenges and opportunities for aggregating information on systemic risks from multiple stakeholders
Challenge mapping
Basadur & Goldsby 2016
Maybe later
Discard
Ginige et al. 2018
No
Discard
Brownfield development challenge categorisation
Fernandes et al. 2020
Yes
Use
Similar context, easy to fit in the questionnaire
Problem tree for challenge mapping
Månssen 2018
No
Discard
Adapted for questionnaire
Q 2: Explain what type of stakeholder you represent. Select from the list below. More than one selection is possible.
Stakeholder categorisation (Rizzo et al 2015)
Group 1: Site owner
Group 2: Authorities
Group 3: Problem holders
Group 4: Service providers
Site owner
Local/city government
Regional/national government
Controlling authorities
Landowner in the surroundings
Local business/other activities
Civil society groups/NGOs/not for profit organisations
Local resident
User of the greenspace
Site developer
Other
Crosby Model about interest and resources
Adapted for questionnaire
Q 4: Please rate your interest in the selected urban greenspaces using the following numbers:
Q 5: Please describe how often you would use the selected urban greenspaces if realised, using the following descriptions:
Q 7: Our development of the green land uses, like any other land use, depends on several types of resources, e.g. time, money, knowledge. Which resources could your organisation potentially contribute with for developing each of green land uses you are interested in?
Brownfield development challenge categorisation
Adapted for questionnaire
Q 8: In your opinion, what are the challenges in developing the site as a prospective urban greenspace? Please describe in the comment box below.
Output (case specific)
Identified and classified stakeholders
Local community
Everyone
Local residents
Students of the residential facilities around
Schools and research institutes
Lindholmen science park
Local businesses
Event organisers
Cafes and restaurants
Gyms nearby
Real estate companies
Student housing authorities
Property owners and developers of the surroundings
The School Lunch Academy
Homeowners
Karlastaden
Älvstranden Utveckling AB
People working in the area
passerby
tourists
non-local residents
Youth
Non-local residents/visitors
Voluntary Nature Conservation
Civil society /NGO
Cultural associations
Färjenäs Forest Garden
Urban Agriculture
Landowner
Government
Real Estate Office
City Planning authority
Recycling and Water Office
Parks and Nature Administration
Environmental Administration
Municipal/ Local government
The County Administrative Board, Västra Götaland
Regional government
Controlling authorities
Mapped interest and resources
UGS Preference
Individual Stakeholders
Government
Local community
Non - local residents (1)
Everyone (31)
Other
Dog park
Outdoor exercise facilities
Collaboratory school garden
Flower meadow
The thickness of the lines represents the number of respondents that selected a specific UGS
Stakeholders (Number of respondent in bracket)
Potential Resources
Municipality
Everyone (31)
Local residents (18)
Time
Knowledge
Money
Time and knowledge
Time, knowledge, and money
Knowledge and money
*Thickness of the connecting lines increase with the number of selection
Identified challenges
Governance
Land
Finance
Design
Sustainability
G1 Co-ordinated planning between administrations
G2 Lack of speed in development due to logistical complicacy and delays
G3 Ensuring maintenance (e.g. withstanding large number of visitor, damage with time, littering, etc.)
G4 Planning restriction on land use
L1 Present derelict condition of the site
L2 Ownership of the land
L3 High density of the locality
L4 Ongoing infrastructure projects around the site
L5 Higher risk of accidents connected to transportation of dangerous goods
L6 Low connectivity to smaller roads and walkways
F1 Economic/financing difficulties
F2 Lack of resources for site development
F3 Potential policy conflict that can lead to large cost in future
D1 Proportionate design of the site to fit the scale of the surroundings
D2 Lack of knowledge and acceptance
D3 Achieving both functionality and aesthetics
D4 Stormwater management with regard to the correct dimensioning
D5 Negatively affecting the possibility of designing a pleasant green area
S1 Ensuring sustainability in exploitation economics
Preferring alternative more income generating land use
S2 Strict competition over land with other plausible land use
Green land use vs other more economically viable alternative such as residence or parking
S3 Present soil contamination
affecting the possibility of designing the green area for cultivation and residence
S4 Noise pollution
S5 Air pollution
Proximity to a road and a railway (Hamnbanan and Lundbyleden)
Overlapping challenges with available resources
Challenges
Stakeholders
UGS realisation phases and resources
Planning phase
Design phase
Building phase
Built phase
Use and maintenance phase
Real Estate office
Parks and Nature Administration
City Planning Authority
Environmental Administration
Älvstranden Utveckling AB
Recycling and Water Office
The County Administrative Board
Students living in the local housing
Local residents/ users of the greenspace today
Time, knowledge, money, access to network of competent designers
Human resources in the form of working hours, expertise, project management, construction management, Management of the completed UGS*
Time for planning and knowledge
Planning documentation and knowledge
Experience in developing parks in the area
Knowledge on rainwater retention
Management and maintenance of UGS
Knowledge
Participate in the design of functions / design, local knowledge
User of UGS
Time, local knowledge
