# Supplementary material for: Transforming brownfields into urban greenspaces: A working process for stakeholder analysis
Source: PLoS One. 2023 Jan 5;18(1):e0278747. doi: 10.1371/journal.pone.0278747 (PMC9815653; doi:10.1371/journal.pone.0278747)
Supplement: S2 File — (DOCX) [file pone.0278747.s002.docx]

**Online questionnaire survey to understand urban greenspace use preference among local stakeholders**

*If you prefer to answer online you can do so with this link <https://www.surveymonkey.com/r/MTXY25B>*

# **Introduction**

Good day! We are a research group at Chalmers investigating the prospects of developing urban greenspaces (UGS) in derelict and abandoned sites in and around Gothenburg. We would be very grateful if you could spend approximately 10 minutes to respond to this questionnaire. It is intended to collect data to better understand preferences regarding various green land uses at a site. The survey is conducted as part of a PhD project at Chalmers University of Technology and the survey results will be reported in a scientific article. The results will not be presented in a way that would allow any individual to be identified. If you have any questions, please contact Shaswati Chowdhury at the Department of Architecture and Civil Engineering (ACE), by e-mail ([shaswati@chalmers.se](mailto:shaswati@chalmers.se)) or by phone (0708240704).

**Case study**

The questions are related to the Polstjärnegatan site – marked in green in the figure below – which is located in Gothenburg in the Karlastaden area of the Lindholmen district. Karlastaden is undergoing rapid transformation to mixed land uses: offices, housing, kindergartens and schools, roads and green spaces. The planned future use of the site is as a park area, specially designed to help with surface water runoff by using bioswales (see explanation further down in the questionnaire). Roads with intensive traffic are passing along this site.


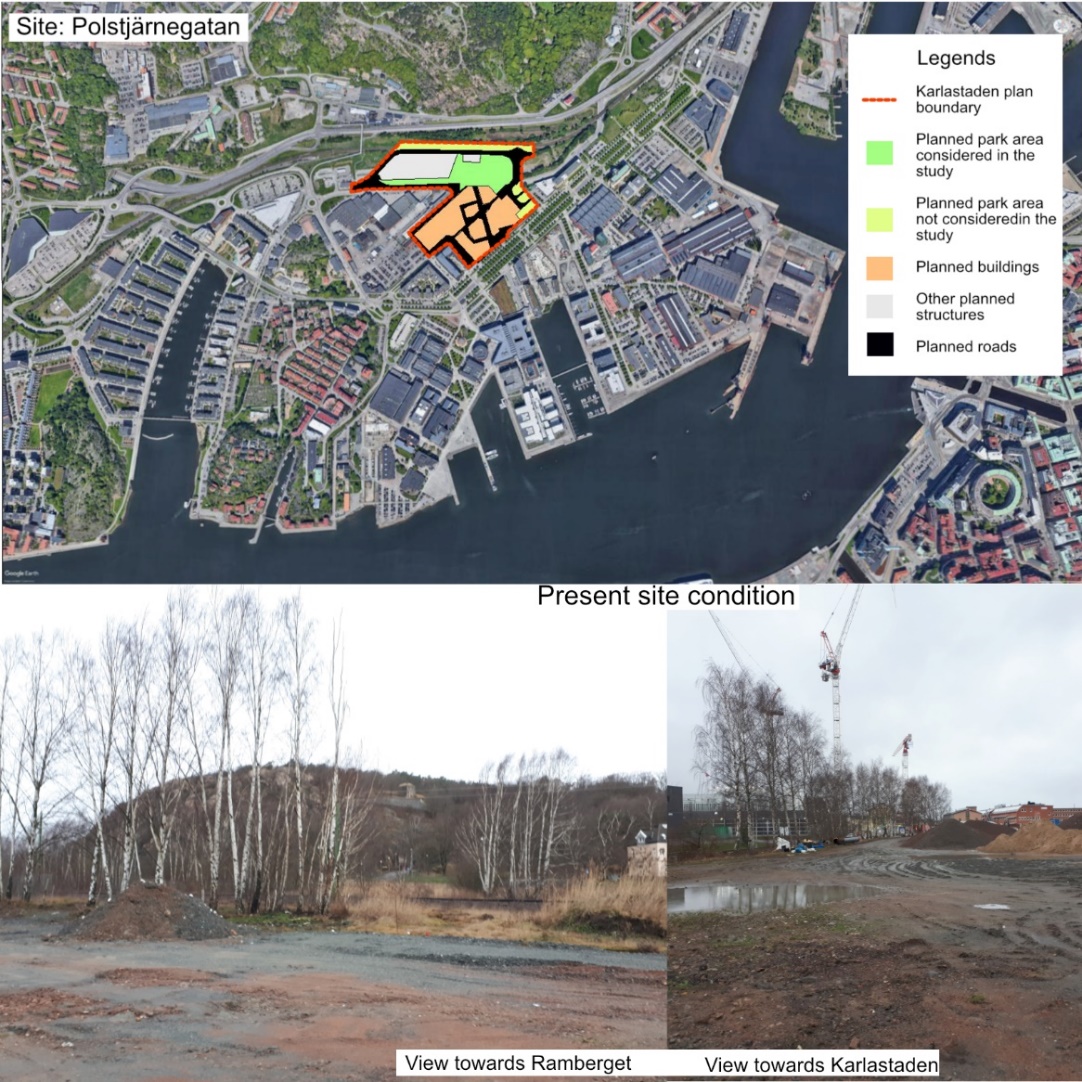


1. What kind of urban greenspace (UGS) you would prefer on the site? Select three from the table below. Explanations for the different types of UGS are given in the table below.


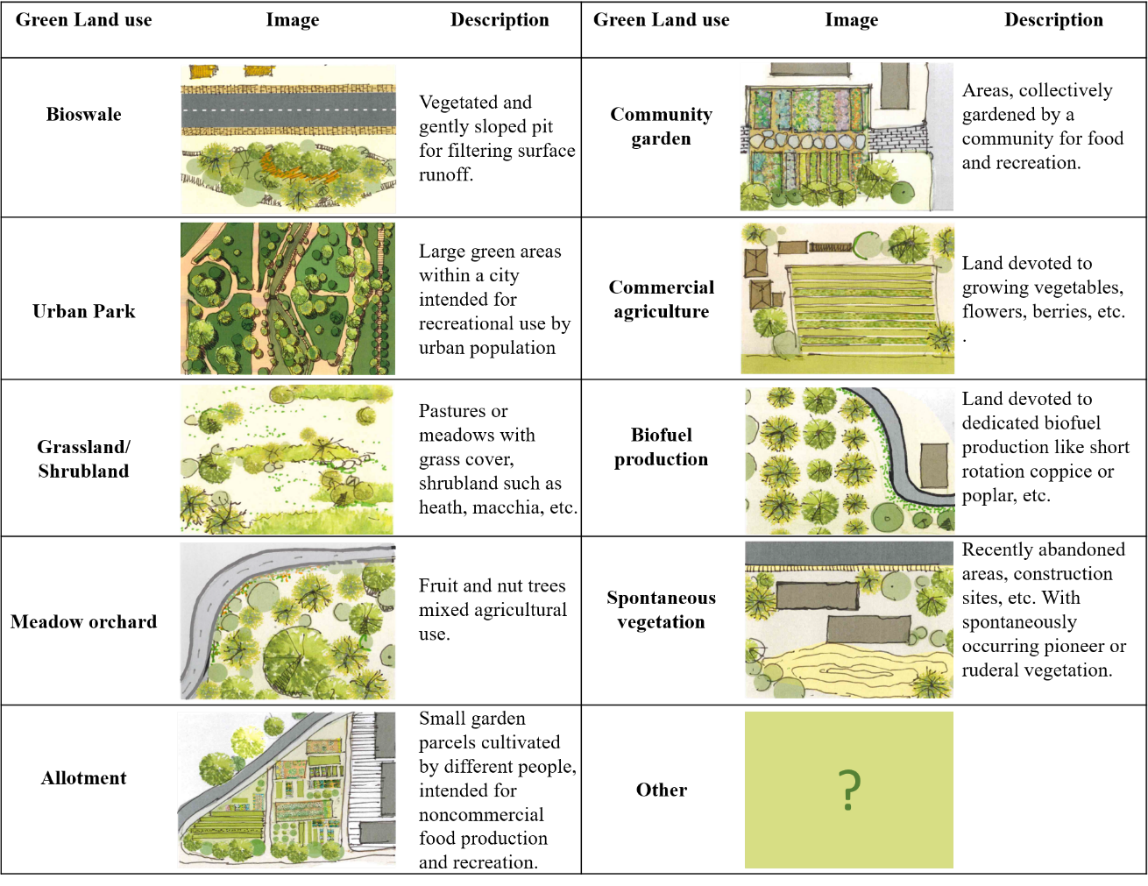


- Bioswale
- Urban park
- Grassland/shrubland
- Meadow orchard
- Allotment
- Community garden
- Commercial agriculture
- Biofuel production
- Spontaneous vegetation
- Other (Please specify)

1. Explain what type of stakeholder you represent. Select from the list below. More than one selection is possible.

- Site owner
- Site developer
- Local/city government
- Regional/national government
- Controlling authorities
- Landowner in the surroundings
- Local business/other activities
- Civil society groups/NGOs/ not for profit organizations
- Local resident
- User of the greenspace today
- Other (please specify)

1. Explain briefly why you are interested in the selected urban greenspace. Only write for your selections of Question 1

Bioswale

Urban park

Grassland/shrubland

Meadow orchard

Allotment

Community garden

Commercial agriculture

Biofuel production

Spontaneous vegetationOther

1. Please rate your interest in the selected urban greenspaces using the following numbers: 0 = no interest; 1 = low; 2 = medium; 3= high. Only rate your selections of Question 1.

Bioswale

Urban park

Grassland/shrubland

Meadow orchard

Allotment

Community garden

Commercial agriculture

Biofuel production

Spontaneous vegetation

Other

1. Please describe how often you would use the selected urban greenspaces if realised, using the following descriptions: 0 = Never; 1 = Sometimes (Once or few times per year); 2 = Often (Several times per year); 3 = Very often (Several times per month). Only write for your selections of Question 1.

Bioswale

Urban park

Grassland/shrubland

Meadow orchard

Allotment

Community garden

Commercial agriculture

Biofuel production

Spontaneous vegetation

Other

1. Development of the green land uses, like any other land use, depends on several types of resources, e.g. time, money, knowledge. Which resources could you/your organisation potentially contribute with for developing each of green land uses you are interested in? Only write for your selections of Question 1.

Bioswale

Urban park

Grassland/shrubland

Meadow orchard

Allotment

Community garden

Commercial agriculture

Biofuel production

Spontaneous vegetation

Other

1. Who else do you think would be interested in developing urban greenspaces on the site? Suggest potential stakeholders that would be relevant/interested in each selected land use. Only write for your selections of Question 1.

Bioswale

Urban park

Grassland/shrubland

Meadow orchard

Allotment

Community garden

Commercial agriculture

Biofuel production

Spontaneous vegetation

Other

1. In your opinion, what are the challenges in developing the site as a prospective urban greenspace? Please describe in the comment box below.
2. If you have something more to say, please write it in the box below.
3. We would appreciate your contact information for the possibility to pose follow-up questions if needed. You may of course also stay anonymous if you wish to do so.

Name

Company

Address

Email address
