# Supplementary material for: Transforming brownfields into urban greenspaces: A working process for stakeholder analysis
Source: PLoS One. 2023 Jan 5;18(1):e0278747. doi: 10.1371/journal.pone.0278747 (PMC9815653; doi:10.1371/journal.pone.0278747)
Supplement: Attachment — Submitted filename: Responses to review comments.docx [file pone.0278747.s004.docx]

PONE-D-22-04756

Transforming brownfields into urban greenspaces: A working process for stakeholder analysis (revised from: Transforming brownfields into urban greenspaces: Development and application of a multi-method approach for Stakeholder analysis)

PLOS-ONE

Dear Editor,

We have revised the article PONE-D-22-04756 in accordance with the changes suggested by the editor and the reviewers. We have explicitly addressed the specific comments of the reviewers, see details in Table 1. The comments were constructive and helped to improve the manuscript.

Best regards,

The authors

# Editor’s summary points

In your response to the reviewer comments, please be sure to address the minor issues brought up by reviewer #2 as well as the more significant issues about the questionnaire pointed out by reviewer #4.

# Reviewers’ comments

Color coding of how the points raised has been addressed:

Completely, Partially, Denied

## Reviewer 2

I carefully went through the manuscript “Transforming brownfields into urban greenspaces: A working process for stakeholder analysis” a second time and can confirm that the text has improved significantly. Most comments have been addressed in a very thorough manner and the authors have done a good job in reframing the text and reworking the figures which are now much clearer and pleasant. Especially the research questions and the methodology are now more concise and link better with the results of the study.

General response:

Thank you for your insights and reflections on the revised texts. Your comments on the original manuscript have been fundamental in restructuring the text and we are very grateful for such through guidance.

*Table 1. Measures and responses to reviewer 2’s comments. The reviewer’s reference to lines in the manuscript are to the original manuscript.*

|  | **Comments** | **How it is addressed (some comments are descriptive and does not require action, so those were marked ‘partially’ as well)** |
| --- | --- | --- |
| Major issues | However, I still suggest further work on the text, mostly in the results section. I acknowledge that the manuscript has been shortened as compared to the earlier version, but it is still unreasonably long, especially given the limited actual evidence in terms of respondents in the survey. The kindly provided tracked changes reveal that relatively little has changed in the results section compared to the earlier version. Therefore, I again suggest some shortening, especially in sections 3.1 and 3.2. These parts are very descriptive, and the contents do not justify such a length that is hard to follow for someone who is not very familiar with this particular case study. Interestingly, the Discussion section now includes a well-written bulleted section (l. 571 ff) summarizing the “main insights”, which is essentially a duplication of the results. I suggest removing this concise summary of the results from the discussion and merging it with the actual results section. This process of rewording should help shortening the results significantly which will help making the overall paper more accessible and meaningful. Instead of describing all stakeholder with all detail, I would rather suggest using more actual quotes from the qualitative part of the surveys, which could help making the text more engaging to read. | We appreciate the constructive feedback, and we also acknowledge that the length of the paper might be challenging. Still, as the article reports on a composite methodology and its application on a case, we feel that the length is justified, and we consider the narrative and descriptive sections as part of the “storytelling” of the paper. In consequence, we also believe that summarizing the main insights in the discussion is helpful for those not delving into the whole paper. Therefore, we have chosen not to follow this suggestion. |
| Minor issues | L 32: challenges with greenspace implementation? | Added as suggested. |
|  | L 35: add type of questionnaire (qualitative, quantitative) and number of respondents | The questionnaire has both qualitative or open ended (e.g. Q3 Explain briefly why you are interested in the selected urban greenspace) and quantitative or close ended (e.g. Q4 Please rate your interest in the selected urban greenspaces using the following numbers: 0 = no interest; 1 = low; 2 = medium; 3= high). So, fixing a type wouldn’t do the questionnaire justice and both are added. The number of respondents is added. The sentences are modified: ‘The methods were applied to a study site in Sweden, and data was collected using a questionnaire survey with both open and close-ended questions. The survey received 31 responses and the respondents’ comments indicated that the combination of several uses…’ |
|  | L 37: remove «effectively» | Removed as suggested. |
|  | L76: remove “of” | Removed as suggested. |
|  | L 76: I guess there are other limitations than just contamination (accessibility, ownership, …) so maybe replace “largely” with “for example” | Edited as suggested. |
|  | L 127: For consistency change wording to “identifying and categorising” or “identification and categorisation” | Edited as suggested. |
|  | L154-155: Not sure if this last sentence is needed. | Has been changed into:  The full complexity of the final Gigamap produced in this study can be appreciated in the attached supplementary material 1. |
|  | Fig 3 and elsewhere: I am a bit unsure about the “Everyone” category. Can this category be renamed to what can actually be considered the full population relevant for this study? Maybe the inhabitants of the city or region? | The category is named reflecting on the respondents’ comments. In a frank way, several respondents explained how they think ‘everyone’ should be involved in realising UGS and we want to capture that essence. But we understand the need for further information and the this line in the text clarifies (L 305-307): The proposed categorisation has one main category (*everyone*) which then includes three cluster categories (*government*, *local community*, *non-local community/visitors*), where all stakeholders that are relevant for this study can appear in one or more clusters. |

## Reviewer 4

This article develops an integrated stakeholder analysis method for urban green space realisation from urban brownfield using a visualisation tool. The application of method is demonstrated by a case study Polstjärnegatan in Gothenburg. This is an interesting topic to explore given the strong demand for green spaces after the pandemic.

*Table 2. Measures and responses to reviewer 3’s comments. The reviewer’s reference to lines in the manuscript are to the original manuscript.*

|  | **Comments** | **How it is addressed (some comments are descriptive and does not require action, so those were marked ‘partially’ as well)** |
| --- | --- | --- |
| Major issues | The authors could justify the advantage and disadvantage of applying the questionnaire survey as the stakeholder analysis method compared to other methods like interview, focus group discussion and workshop, as it is a new approach applied to understand stakeholder interests (Section 2.3, line 199-204). For example, questionnaire is potentially a useful strategy to collect different views for regeneration cases | The following sentence has been added:  A questionnaire survey for data collection also meant that respondents could answer at their own convenience and being largely online and anonymous, helped the respondents to be frank with their answers (Williamson, 2018). |
|  | A total of 31 responses are collected, while 18 of them are from local students and local residents. Students could be important stakeholder but they may not be long-term residents in the area, so I wonder if they could represent the community opinion. The feedbacks from other stakeholders (like local business) are limited and maybe not enough to show the interest of whole stakeholder group. It would be helpful to add a bit more site context about the general local community component and distribution (e.g. local demographics, how many residence estates vs. student facilities). Otherwise it would be hard to determine whether the collected samples are representative enough. | Students form a large part if the inhabitants in this area and will continue to do so. To make this clear, the following text has been re-formulated:  The study site at Polstjärnegatan is located within the Lindholmen district in Gothenburg (Sweden). Population of Lindholmen is around 5000 according to 2022 census but more than 10000 students come to study here due to the district being an education hub with several schools and a campus of Chalmers University of Technology (Göteborgs stadsledningskontor, 2022; Lundby stadsdelsförvaltning, 2020). Lindholmen district has less than 3000 housing units at present and a significant part of them are student apartments (nearly 700 registered apartments by the student housing associations alone) (Göteborgs stadsledningskontor, 2022; SGS Studentbostäder, 2022). The site is part of the concept plan of Karlastaden, a large-scale housing and commercial facility (Fig. 2, top), whose construction (as of 2022) is in progress (Göteborgs Stad, 2017, 2022). The development is projected to finish by 2026 and will add 2000 more apartments to the Lindholmen area (Göteborgs Stad, 2017, 2022). The site has confirmed contamination issues due to previous uses (Kaltin & Almqvist, 2016). It is surrounded by roads and a railway on all sides: Polstjärnegatan to the south, Karlavagnsgatan to the east, a petrol station and a fast-food restaurant to the west, and a railway (Hamnbanan) as well as a motorway (Lundbyleden) to the north (see Fig. 2). |
|  | I wonder how identified challenges in this study are associated with broader context of brownfield-UGS regeneration in the country. Conflicts of interest (mentioned in line 113 in introduction) seems to be a key challenge discussed in the literature, while the selected case study does not directly demonstrate this potential challenge. The discussion on what insights can be learned from the case study to inform similar cases in the future seems to be insufficient (Section 3.3 and line 589-592 in Section 4). | Thank you for pointing this out. The following paragraph has been added in the discussion:  In a review on the challenges associated with UGS planning in cities, Haaland & van den Bosch (2015) confirms that densification processes such as infill development and consolidation can pose a threat to UGS. The site in this study is in a rapidly developing and densifying urban district and is part of a development project, Karlastaden, that consists of eight urban blocks of mixed commercial and residential development (cite). Even though the site is designated to be designed as a greenspace, it can be considered as part of a larger urban densification project. With the speed of urbanisation in the area, the concern of the local stakeholders (Challenge S1 and S2 in Fig 6) that greenspaces in the area can come under more pressure to be repurposed for more economically beneficial (e.g. housing or commercial) land use. The dense Karlastaden comes with another challenge is that it consists of four high-rises and impact of such in planning the greenspace. Although unique to the local context, high-rise neighbourhoods exist in many cities across the world and the design challenges are studied for different contexts (FFLA, 2018; Ye et al., 2020). There are now more subtler challenges, as explained by Colding et al. (2020), that can conjugate and pose a greater threat in realising and maintaining UGS in cities. Challenges regarding ensuring maintenance (Challenge G3 in Fig 6) that stems from lack of financial resources (Challenge F1 and F2) can potentially lead to outsourcing the management to private sector. Such challenges as observed by the stakeholders at the study site are argued by Colding et al. (2020) to result in gradual loss of access to greenspaces across many cities due to privatisation. This can negatively affect the urban inhabitants, specifically for citizens in Swedish cities, who largely associate their well-being with access to natural areas (Elbakidze et al., 2017) and make frequent visits to different greenspaces located within the cities (Rydberg & Falck, 1998). Even with almost 70% of land area covered with forest in Sweden (SLU, 2022), more than 50% of forest visits happen in the urban forests with most frequent visits occurring in the ones located withing 1 km of the residents’ home (Kardell, 1985; Rydberg & Falck, 1998). At the study site, the respondents also emphasised on the importance of UGS by reasoning how stakeholders across all domains potentially benefit from them. The challenges identified for UGS realisation by the respondents as well as the resources they bring to tackle the challenges can help to support UGS realisation and maintenance in cities. |
| Specific issues | Line 213: can add a bit more detail about the ten UGS types used in the questionnaire (for example, with a reference to UGS classification), and describe other questions enquired in the questionnaire a bit (1-2 sentences). | These lines are added: ‘The list of possible UGS at the study site and description of these UGS in the questionnaire was based on Chowdhury et al. (2020), using the classification/categorisation of the Greensurge project (Haase et al. 2015). The UGS presented in the questionnaire were: bioswale, urban park, grassland/shrubland, meadow orchard, allotment, community garden, commercial agriculture, biofuel production, and spontaneous vegetation.’ |
|  | Figure 7 (bottom). ‘Human resources in the form of work neighbours…’ needs a space between ‘resources’ and ‘in’. | Done |
